# Mapping epigenetic modifications on chicken lampbrush chromosomes

**DOI:** 10.1186/s13039-020-00496-0

**Published:** 2020-08-03

**Authors:** Tatiana Kulikova, Anna Surkova, Anna Zlotina, Alla Krasikova

**Affiliations:** Saint Petersburg State University, Saint-Petersburg, Russia

**Keywords:** Chromatin domain, Chromomere, Cytological chromomere-loop map, Chicken, Epigenetic modifications, Gene mapping, Histone modifications, Karyotype, Lampbrush chromosomes, Methylated cytosine, Tandem repeats

## Abstract

**Background:**

The epigenetic regulation of genome is crucial for implementation of the genetic program of ontogenesis through establishing and maintaining differential gene expression. Thus mapping of various epigenetic modifications to the genome is relevant for studying the regulation of gene expression. Giant transcriptionally active lampbrush chromosomes are an established tool for high resolution physical mapping of the genome and its epigenetic modifications. This study is aimed at characterizing the epigenetic status of compact chromatin domains (chromomeres) of chicken lampbrush macrochromosomes.

**Results:**

Distribution of three epigenetic modifications – 5-methylcytosine, histone H3 trimethylated at lysine 9 and hyperacetylated histone H4 – along the axes of chicken lampbrush chromosomes 1–4, Z and W was analyzed in details. Enrichment of chromatin domains with the investigated epigenetic modifications was indicated on the cytological chromomere-loop maps for corresponding chicken lampbrush chromosomes. Heterogeneity in the distribution of 5-methylcytosine and histone H3 trimethylated at lysine 9 along the chromosome axes was revealed.

**Conclusions:**

On examples of certain chromomeres of chicken lampbrush chromosomes 1, 3, 4 and W we demonstrated that a combination of immunofluorescent staining and fluorescence in situ hybridization allows to relate the epigenetic status and a DNA sequence context of individual chromomeres.

## Background

Lampbrush chromosomes are highly extended transcriptionally active chromosomes that appear at the diplotene stage of meiotic prophase I in growing oocytes of all vertebrate taxons, except mammals. Lampbrush chromosomes have a prominent chromomere-loop organization [1–3]. Condensed chromatin is accumulated in chromomeres – deoxyribonucleoprotein granules about 1 μm in size, connected in a chain by thin axes of chromatin [4, 5]. Average length of genomic segment packed into a single lampbrush chromomere is estimated as 1.5-2 Mb for chicken [6] and 5–10 Mb for urodele amphibians [5]. Transcriptionally active chromatin is organized in paired lateral loops outgoing from the chromomeres. Being highly decondensed and enriched with morphological markers, lampbrush chromosomes represent a promising tool for high-resolution physical gene mapping [6]. Individual genes or tandem repeat families can be mapped precisely to certain lampbrush chromomeres or lateral loops by fluorescence in situ hybridization (FISH) according to DNA/DNA and/or DNA/RNA hybridization protocols [7]. Moreover, lampbrush chromosomes allow to investigate the pattern of various epigenetic modifications in both completely decondensed (lateral loops) and condensed (chromomeres) chromatin domains.

General distribution of certain epigenetic modifications on avian and amphibian lampbrush chromosomes has been earlier described. Notably, lampbrush chromosomes lack linker histone H1 [8]. Essential marker of transcriptionally active chromatin – hyperacetylated histone H4 (H4Ac) – was revealed in the axes of lateral loops, areas of contacts of lateral loops with chromomeres as well as in a chromomere core [3, 9]. 5-methylcytosine (5mC) was found to be accumulated in chromomeres and in untranscribed regions of lateral loops [2, 10, 11]. Other markers of transcriptionally inactive chromatin (histone H3 trimethylated at lysine 9 (H3K9me3) or lysine 27 (H3K27me3)) accumulate in the regions of constitutive heterochromatin such as pericentromeric and subtelomeric chromomeres, polymorphic segments of chromosomes [12] and sex chromosome W [11]. All chromatin modifications studied on lampbrush chromosomes demonstrate more or less heterogeneous distribution along the chromosome axes with the exception of histones H4 and H2A phosphorylated by serine 1, which quite homogeneously enrich the majority of lampbrush chromomeres [11].

To date there are no maps illustrating the distribution of epigenetic modifications along the axes of lampbrush chromosomes. The aim of this study was to develop working chromosome maps reflecting the enrichment of 5mC, H3K9me3 and H4Ac in certain lampbrush chromomeres. Lampbrush chromosomes of domestic chicken (*Gallus gallus domesticus*) were used as a model for studying the epigenetic status of chromomeres. Genome of domestic chicken is almost completely deciphered [13]. Moreover, cytological chromomere-loop maps reflecting the number and size of chromomeres, the intensity of DAPI-staining of chromomeres, the average length of lateral loops in a region as well as the positions of centromeres and marker structures were designed for all chicken macrochromosomes [6, 14, 15] and the largest microchromosomes in a lampbrush form [16]. Certain number of tandem repeats and BAC clones was mapped on chicken lampbrush macrochromosomes [6, 14–20]. It is important to note that individual chromomeres can be microdissected to generate chomomere specific FISH-probes. Furthermore the obtained DNA samples are applicable for sequencing enabling to define genomic position of a chromomere and to analyze its DNA context [21, 22].

Here we mapped the pattern of chromomeric distribution of three epigenetic modifications (5mC, H3K9me3 and H4Ac) to corresponding cytological chromomere-loop maps of *G. g. domesticus* lampbrush chromosomes (GGA) 1–4, Z and W. In addition we demonstrated that the obtained maps could be applied to relate the DNA sequences of individual lampbrush chromomeres with their epigenetic status.

## Results

**Fig. 1:**
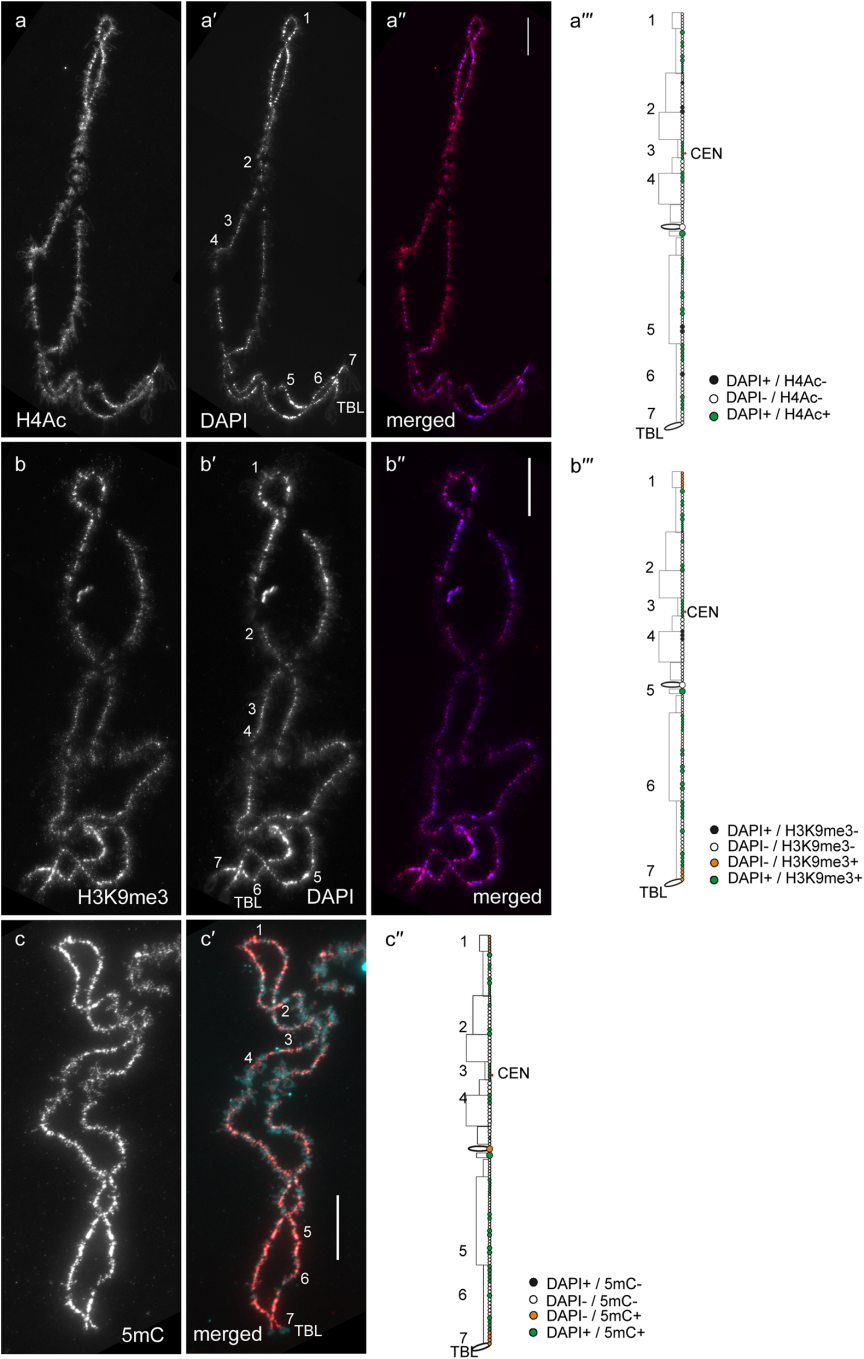
Distribution of H4Ac, H3K9me3 and 5mC on chicken lampbrush chromosome 1 (GGA1). Immunostaining of GGA1 with antibodies against H4Ac (**a**), H3K9me3 (**b**) and 5mC (**c**). **a′**, **b′** – DAPI staining of corresponding chromosomes. **a′′**, **b′′**, **c′** – merged images of corresponding chromosomes (immunostaining – red, DAPI – blue (**a′′**, **b′′**) or cyan (**c′**)). Scale bars – 20 μm. **a′′′**, **b′′′**, **c′′** – maps of corresponding epigenetic modifications distribution. DAPI-positive chromomeres – white circles, DAPI-negative chromomeres – black circles, DAPI-positive chromomeres enriched with an epigenetic marker – green circles; DAPI-negative chromomeres enriched with an epigenetic marker – orange circles. Numbers (1-7) indicate chromomere clusters or chromosome regions described in the Results section. CEN – centromere position, TBL – telomere bow-like loops

**Fig. 2:**
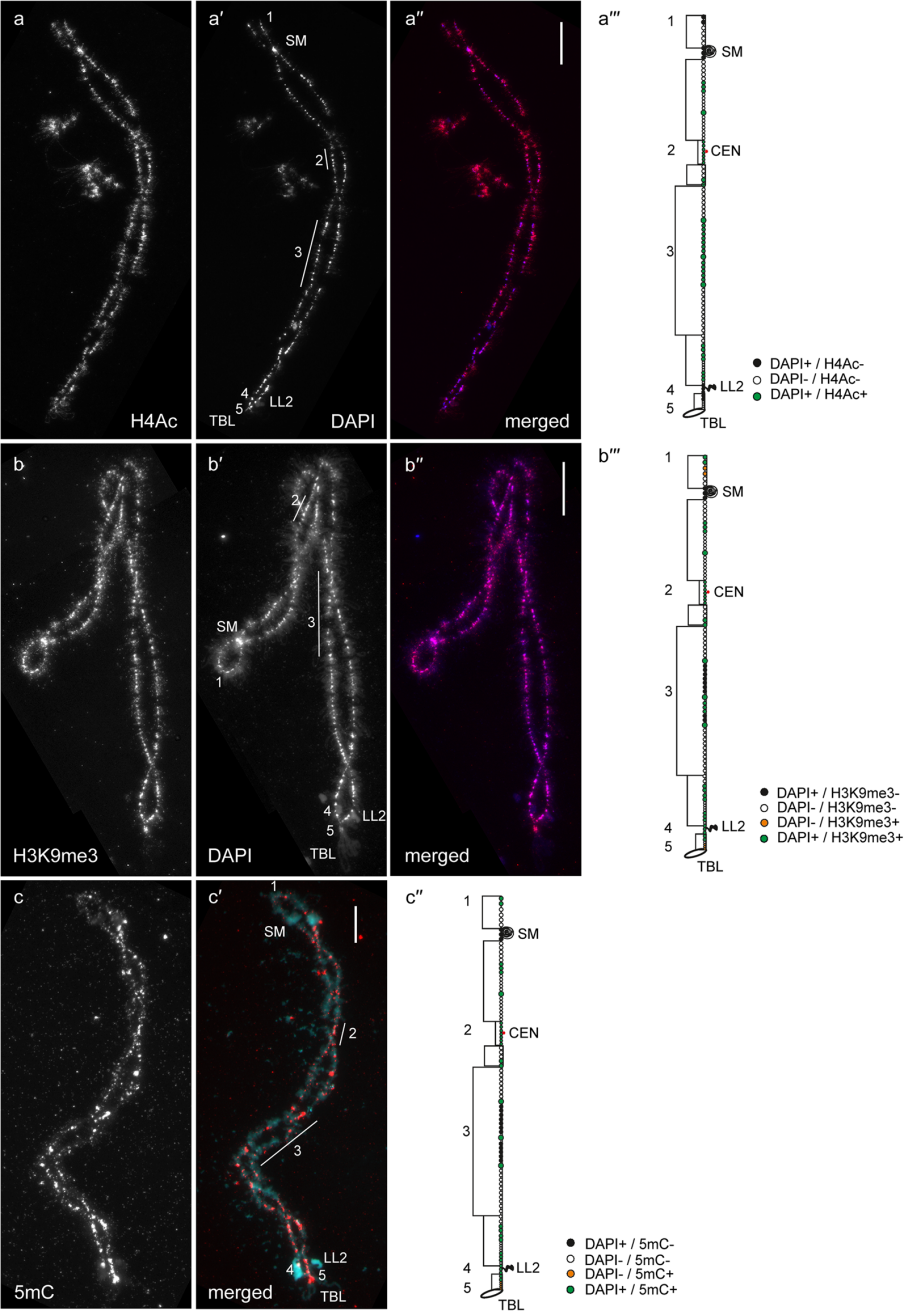
Distribution of H4Ac, H3K9me3 and 5mC on chicken lampbrush chromosome 2 (GGA2). Immunostaining of GGA2 with antibodies against H4Ac (**a**), H3K9me3 (**b**) and 5mC (**c**). **a′**, **b′** – DAPI staining of corresponding chromosomes. **a′′**, **b′′**, **c′** – merged images of corresponding chromosomes (immunostaining – red, DAPI – blue (**a′′**, **b′′**) or cyan (**c′**)). Scale bars – 20 μm.** a′′′**, **b′′′**, **c′′** – maps of corresponding epigenetic markers distribution. Intensity of immunostaining and DAPI-staining pattern are indicated by colors as on Fig. 1**a′′′**, **b′′′**, **c′′**. Numbers and lines (1–5) indicate chromomere clusters or chromosome regions described in the Results section. CEN – centromere position, TBL – telomere bow-like loops, SM – spaghetti marker, LL2 – lumpy loop 2. Staining of the chromomeres with anti-H4Ac and anti-H3K9me3 at the loci of SM formation were not indicated on maps since SM cross-reacts with rabbit serum

**Fig. 3:**
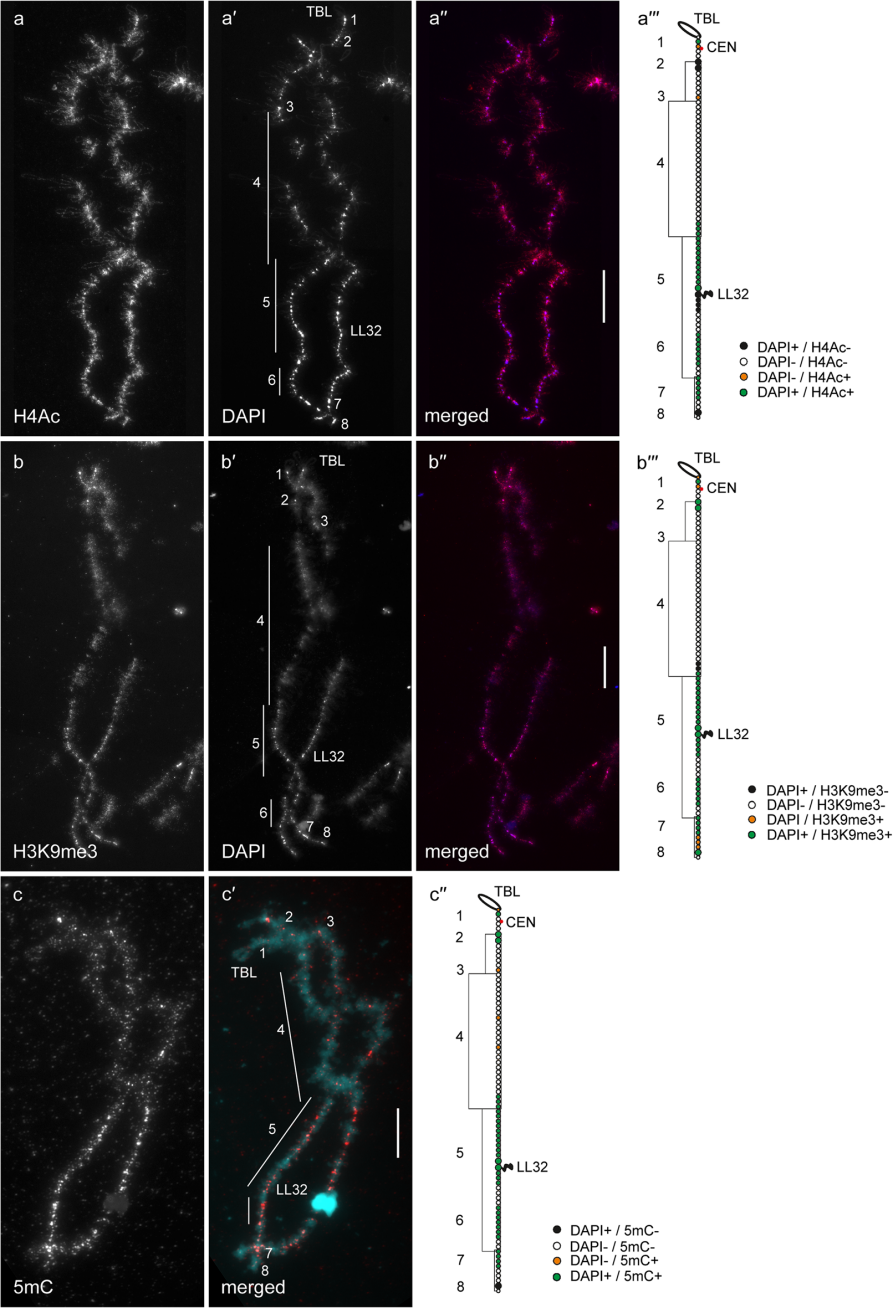
Distribution of H4Ac, H3K9me3 and 5mC on chicken lampbrush chromosome 3 (GGA3). Immunostaining of GGA3 with antibodies against H4Ac (**a**), H3K9me3 (**b**) and 5mC (**c**). **a′**, **b′** – DAPI staining of corresponding chromosomes. **a′′**, **b′′**, **c′** – merged images of corresponding chromosomes (immunostaining – red, DAPI – blue (**a′′**, **b′′**) or cyan (**c′**). Scale bars – 20 μm. **a′′′**, **b′′′**, **c′′** – maps of corresponding epigenetic modifications distribution. Intensity of immunostaining and DAPI-staining pattern are indicated by colors as on Fig. 1**a′′′**, **b′′′**, **c′′**. Numbers and lines (1–8) indicate chromomere clusters or chromosome regions described in the Results section. CEN – centromere position, TBL – telomere bow-like loops, LL32 – lumpy loop 32

**Fig. 4:**
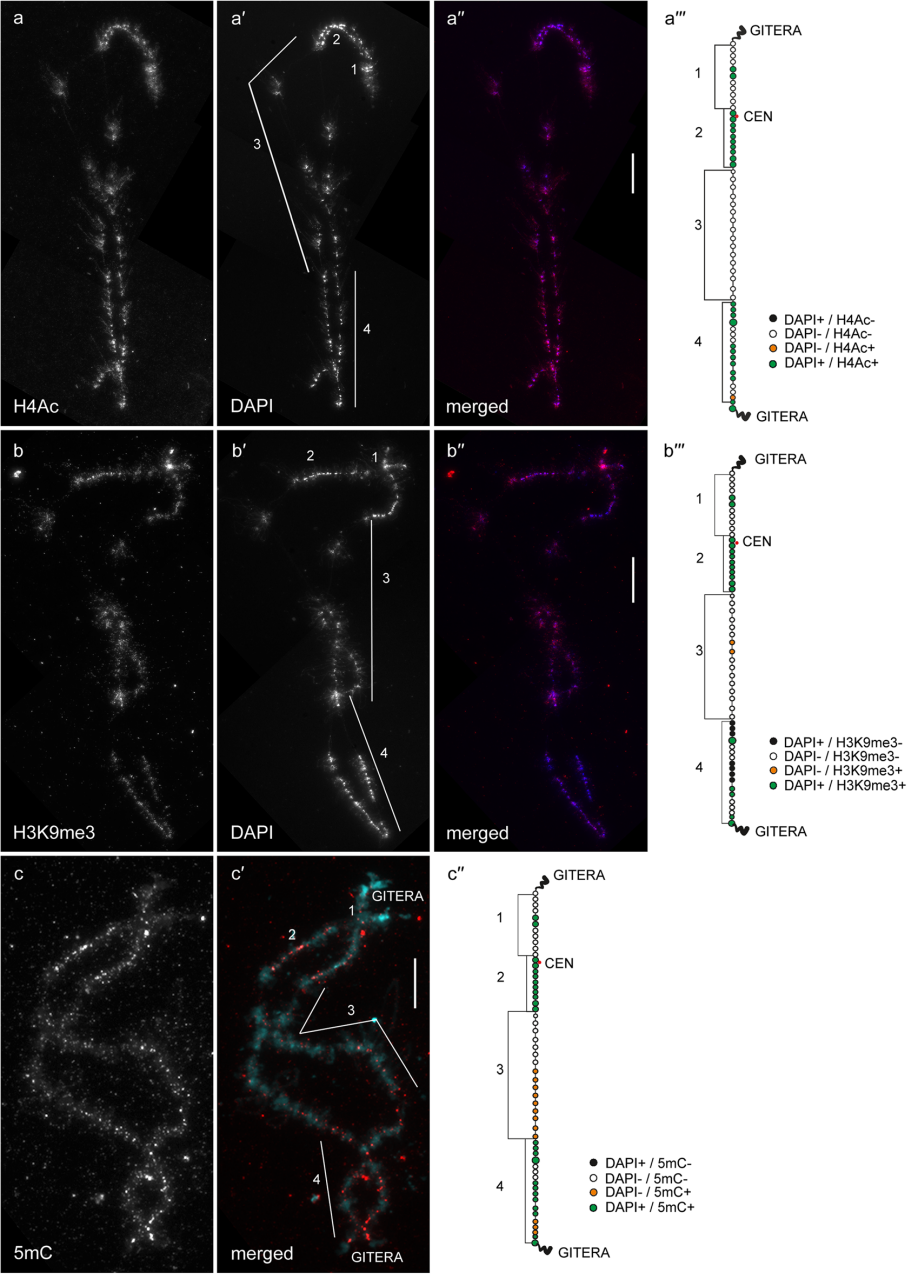
Distribution of H4Ac, H3K9me3 and 5mC on chicken lampbrush chromosome 4 (GGA4). Immunostaining of GGA4 with antibodies against H4Ac (**a**), H3K9me3 (**b**) and 5mC (**c**). **a′**, **b′** – DAPI staining of corresponding chromosomes. **a′′**, **b′′**, **c′** – merged images of corresponding chromosomes (immunostaining – red, DAPI – blue (**a′′**, **b′′**) or cyan (**c′**). Scale bars – 20 μm. **a′′′**, **b′′′**, **c′′** – maps of corresponding epigenetic modifications distribution. Intensity of immunostaining and DAPI-staining pattern are indicated by colors as on Fig. **1****a′′′**, **b′′′**, **c′′**. Numbers and lines (1–4) indicate chromomere clusters or chromosome regions described in the Results section. CEN – centromere position, GITERA – giant terminal RNP aggregates

**Fig. 5:**
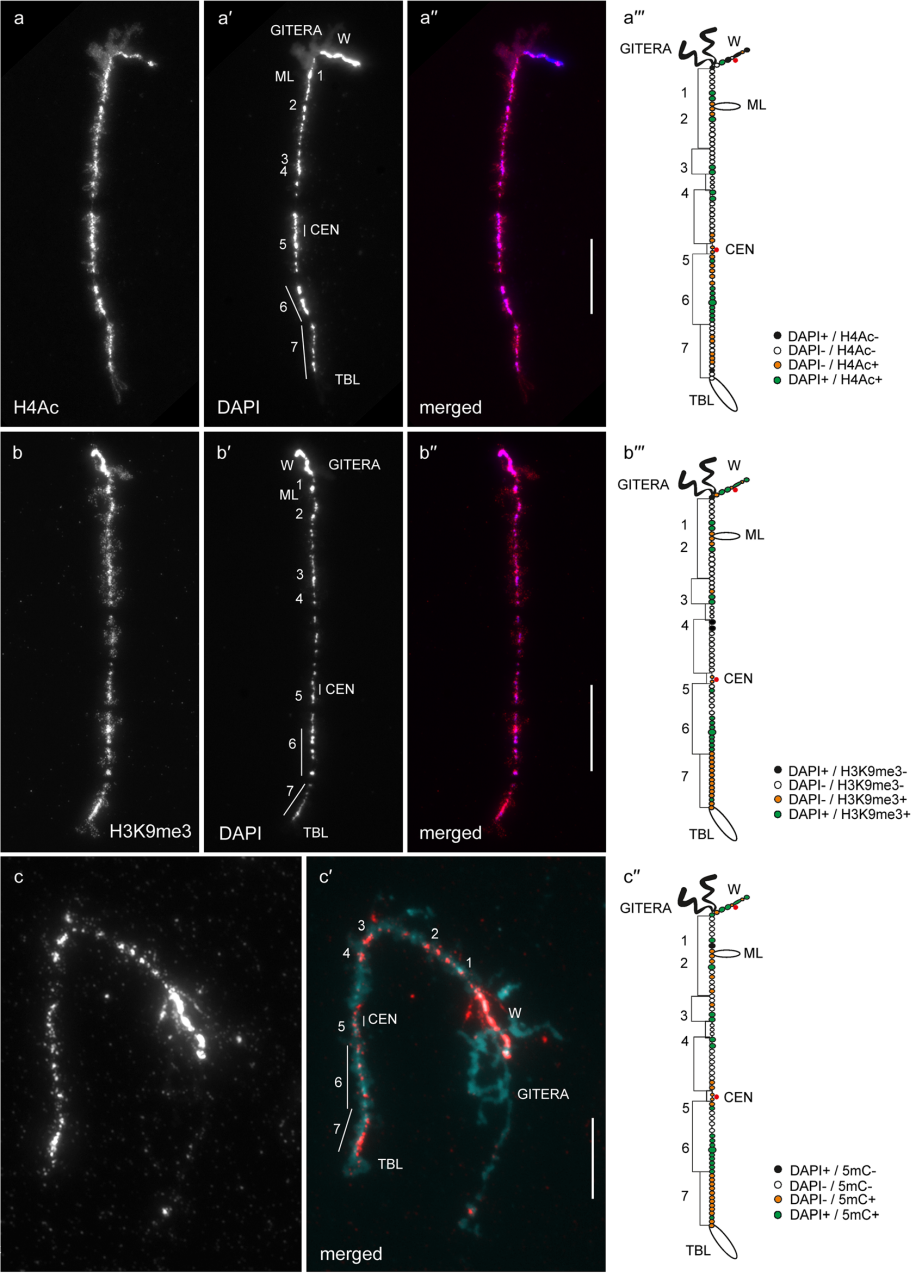
Distribution of H4Ac, H3K9me3 and 5mC on chicken lampbrush chromosome Z (GGAZ). Immunostaining of GGAZ with antibodies against H4Ac (**a**), H3K9me3 (**b**) and 5mC (**c**). **a′**, **b′** – DAPI staining of corresponding chromosomes. **a′′**, **b′′**, **c′** – merged images of corresponding chromosomes (immunostaining – red, DAPI – blue (**a′′**, **b′′**) or cyan (**c′**)). Scale bars – 20 μm. **a′′′**, **b′′′**, **c′′** – maps of corresponding epigenetic modifications distribution. Intensity of immunostaining and DAPI-staining pattern are indicated by colors as on Fig. **1****a′′′**, **b′′′**, **c′′**. Numbers and lines (1–7) indicate chromomere clusters or chromosome regions described in the Results section. CEN – centromere position, ML – marker loop, TBL – telomere bow-like loops, GITERA – giant terminal RNP aggregates

**Fig. 6:**
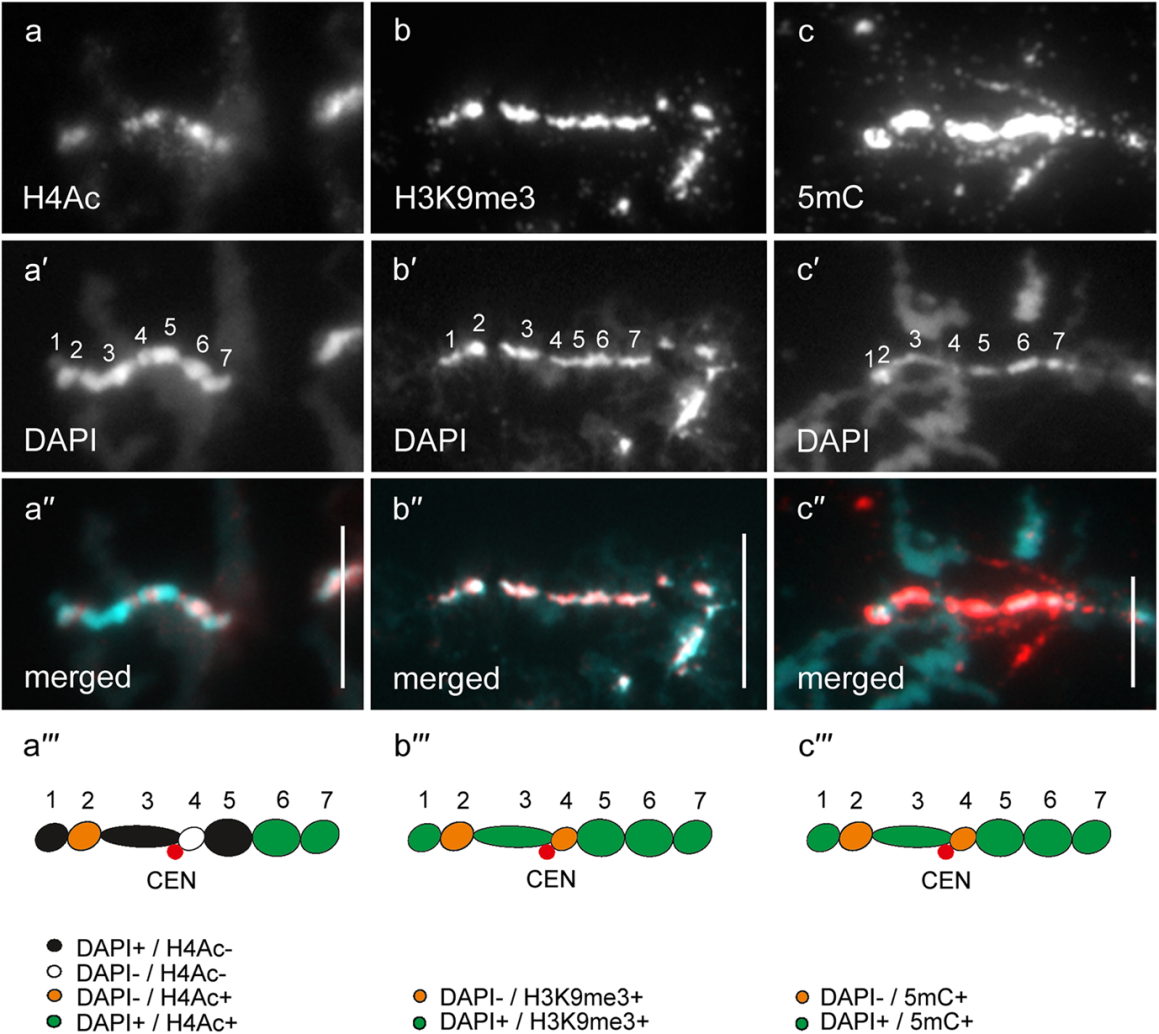
Distribution of H4Ac, H3K9me3 and 5mC on chicken lampbrush chromosome W (GGAW). Immunostaining of GGAW with antibodies against H4Ac (**a**), H3K9me3 (**b**) and 5mC (**c**). **a′**, **b′**, **c′** – DAPI staining of corresponding chromosomes. **a′′**, **b′′**, **c′′** – merged images of corresponding chromosomes (immunostaining – red, DAPI – cyan). Scale bars – 10 μm. **a′′′**, **b′′′**, **c′′′** – maps of corresponding epigenetic modifications distribution. Intensity of immunostaining and DAPI-staining pattern are indicated by colors as on Fig. **1****a′′′**, **b′′′**, **c′′**. Numbers (1–7) indicate individual chromomeres of GGAW from free terminus to chiasma with the chromosome Z. CEN – centromere position

In general, by immunfluorescent staining we revealed predominant enrichment of all three studied epigenetic modifications (H4Ac, H3K9me3 and 5mC) in chromomeres brightly stained with DAPI (hereinafter referred to as DAPI-positive chromomeres) (Figs. 1, 2, 3, 4, 5 and 6). H4Ac demonstrated a punctate distribution pattern on lateral loops and in the areas of contact of lateral loops with chromomeres, which is expected for the maker of an open chromatin. In lampbrush chromosome axes H4Ac was enriched on the surface of certain chromomeres, predominantly DAPI-positive ones. On the contrary, H3K9me3 was nearly undetectable in the axes of lateral loops but was enriched in lampbrush chromomeres. Anti-H3K9me3 antibodies showed a heterogeneous staining pattern along lampbrush chromosome axes with the most bright labeling in the q-terminus of chromosome Z (Fig. 5 b-b′′′) and all chromomeres of GGAW (Fig. 6 b-b′′′). Immunostaining with antibodies against 5mC revealed its enrichment in the majority of DAPI-positive chromomeres and minor content along lateral loop axes as well as at the loop bases. With that, the distribution pattern of 5mC mostly matched the distribution pattern of H3K9me3; vivid examples are chromomeres of GGAW (Fig. 6 b-c′′′). Few chromomeres and/or clusters of chromomeres faintly stained with DAPI (hereinafter referred to as DAPI-negative chromomeres) demonstrated enrichment with one, two or all three epigenetic modifications. The staining pattern was reproducible, reflecting the association of the histone modifications and DNA-methylation with defined genomic regions during the lampbrush stage of oogenesis.

### Cytological maps and description of the distribution of H4Ac, H3K9me3 and 5mC in chromomeres of chicken lampbrush chromosomes 1–4, Z and W

The distribution of H4Ac, H3K9me3 and 5mC along the axes of chicken lampbrush chromosomes 1–4, Z and W was indicated on the corresponding cytological chromomere-loop maps reflecting DAPI-staining pattern and the average length of lateral loops [6, 15]. In the following descriptions certain chromosomal regions, clusters and individual chromomeres are specified by numbers, marked both in microphotographs and maps.

#### GGA1

By immunofluorescent staining we revealed generally similar distribution of H4Ac, H3K9me3 and 5mC on GGA1, which also mostly corresponded to the DAPI staining pattern (Fig. 1). The majority of DAPI-positive chromomeres, including cluster surrounding the centromere (3), were enriched with both H4Ac and H3K9me3 (Fig. 1 a-b′′′). The exclusion were several DAPI-positive chromomeres faintly labeled with anti-H4Ac but brightly labeled with anti-H3K9me3 (2, 5, 6) and a chromomere cluster on the q-arm (4) brightly labeled with anti-H4Ac but faintly labeled with anti-H3K9me3 (Fig. 1 a-b′′′). All DAPI-positive chromomeres of GGA1 demonstrated enrichment with 5mC (Fig. 1 c-c′′). Thus chromomeres combining H4Ac and H3K9me3 modifications were also enriched with 5mC. Interestingly, сlusters of DAPI-negative chromomeres at the termini of GGA1 (1, 7) were enriched with both H3K9me3 and 5mC, but not with H4Ac (Fig. 1 b-c′′). These regions contain neighboring clusters of tandem repeat PO41 [17] and Z-macrosatellite [23].

#### GGA2

As in case of GGA1, relatively bright labeling with anti-H4Ac was observed mostly in DAPI-positive chromomeres of GGA2 (Fig. 2 a-a′′′). The majority of H4Ac- and DAPI-positive chromomeres, including the centromere cluster (2), were also enriched with H3K9me3 and 5mC. The exception was the extended cluster of chromomeres on the q-arm (3) where only large chromomeres demonstrated prominent labeling with anti-H3K9me3 and anti-5mC (Fig. 2 b-c′′). Terminal chromomeres (1 and 5) containing Z-macrosatellite [23], subterminal chromomeres of the q-arm (5) containing PO41 repeat [17] as well as more proximal q-arm region (4) were faintly labeled with anti-H4Ac (Fig. 2 a-a′′′) but brightly labeled with both anti-H3K9me3 and anti-5mC (Fig. 2 b-c′′).

#### GGA3

P-arm of GGA3 consists of only three-four chomomeres (1). The terminal chromomere bearing loops with transcription units of Z-macrosatellite [23] was faintly labeled with anti-H4Ac (Fig. 3 a-a′′′) but brightly labeled with anti-H3K9me3 and anti-5mC (Fig. 3 b-с′′). The next DAPI-positive chromomere was enriched with all three epigenetic modifications. The third chromomere adjacent to a centromere granule [14] was enriched with H4Ac and H3K9me3 but not with 5mC. On the q-arm of GGA3, a pair of large DAPI-positive chromomeres (2) containing non-transcribing cluster of 41 bp tandem repeat CNM [14, 18] were depleted with H4Ac (Fig. 3 a-a′′′) but enriched with both H3K9me3 and 5mC (Fig. 3 b-с′′). At the same time, DAPI-negative chromomere bearing lateral loops with transcription units of CNM repeat (3) [14, 18] demonstrated bright labeling with anti-H4Ac and anti-5mC (Fig. 3 a-a′′′, c-c′′) but moderate labeling with anti-H3K9me3 (Fig. 3 b-b′′′). An extended cluster of DAPI-negative chromomeres (4) was faintly labeled with H4Ac and H3K9me3 (Fig. 3 a-b′′′); 5mC was revealed in some individual chromomeres within the cluster (4) (Fig. 3 c-c′′). Clusters of DAPI-positive chromomeres (5, 6 and 7) demonstrated bright labeling with antibodies against all three epigenetic modifications. The only exception was chromomere bearing LL32 (lumpy loop 32) and several distal chromomeres which were depleted with H4Ac (Fig. 3 a-a′′′) but enriched with H3K9me3 and 5mC (Fig. 3 b-c′′). Terminal region of the q-arm (8) containing minor cluster of Z-macrosatellite [23] demonstrated enrichment with only H3K9me3 (Fig. 3 b-b′′′).

#### GGA4

The majority of DAPI-positive chromomeres of the GGA4 demonstrated bright labeling with anti-H4Ac, anti-H3K9me3, and anti-5mC (Fig. 4). These include DAPI-positive chromomeres of the p-arm (1), cluster of chromomeres surrounding the centromere (2) and terminal clusters of DAPI-positive chromomeres in the region 4 of the q-arm with short lateral loops. The exceptions were two clusters of DAPI-positive chromomeres in the region 4, which were less brightly stained with anti-H3K9me3 (Fig. 4 b-b′′′). The majority of DAPI-negative chromomeres forming an extended cluster on the q-arm (3) were almost depleted with H4Ac and H3K9me3 (Fig. 4 a-b′′′); certain chromomeres in the distal half of this region were enriched with 5mC (Fig. 4 c-c′′). Bright anti-5mC labeling was also revealed in the subterminal cluster of DAPI-negative chromomeres of the q-arm (Fig. 4 b-c′′).

#### GGAZ

A pair of prominent DAPI-positive chromomeres on the distal part of GGAZ p-arm (1) was brightly labeled with antibodies against H4Ac and H3K9me3; 5mC was revealed only in the distal chromomere of the pair (Fig. 5). A following cluster of DAPI-negative chromomeres and a DAPI-positive chromomere (2) demonstrated enrichment with all three epigenetic modifications. The next pair of DAPI-positive chromomeres (3) was also labeled with antibodies against all three epigenetic modifications (Fig. 5). More proximal pair of DAPI-positive chromomeres (4) was brightly labeled only with anti-H4Ac and anti-5mC (Fig. 5 a-a′′′, c-c′′). DAPI-negative chromomeres at the centromere region as well as a proximal DAPI-positive chromomere of the q-arm (5) and a large cluster of DAPI-positive chromomeres (6) were enriched with all three epigenetic modifications (Fig. 5). A cluster of DAPI-negative chromomeres between chromomeres 5 and 6 was enriched only with H4Ac (Fig. 5 a-a′′′). The terminal region of the q-arm occupied by the Z-mascrosatellite (7) was highly enriched with H3K9me3 and 5mC (Fig. 5 b-c′′); certain DAPI-negative chromomeres of this region were also enriched with H4Ac (Fig. 5 a-a′′′).

#### GGAW

Lampbrush chromosome W consists of seven compact chromomeres associated with the clusters of certain tandem repeats [14, 24–26]. All chromomeres of GGAW demonstrated enrichment with H3K9me3 and 5mC (Fig. 6 b-c′′′). Remarkably, we found certain chromomeres enriched with H4Ac as well. These are *Ssp*I-repeat containing chromomeres (2, 6 and a minor cluster at the proximal end of the chromomere 5) [14], centromere region (at the proximal border with the chromomere 3) [14, 21] and chromomere 7 containing a G-rich microsatellite [26] (Fig. 6 a-a′′′). The position of chromomere 4 containing cluster of CNM repeat was verified by FISH with oligonucleotide probe after immunostaining (Supplementary Fig. 1).

### Epigenetic status of individual chromomeres

**Fig. 7:**
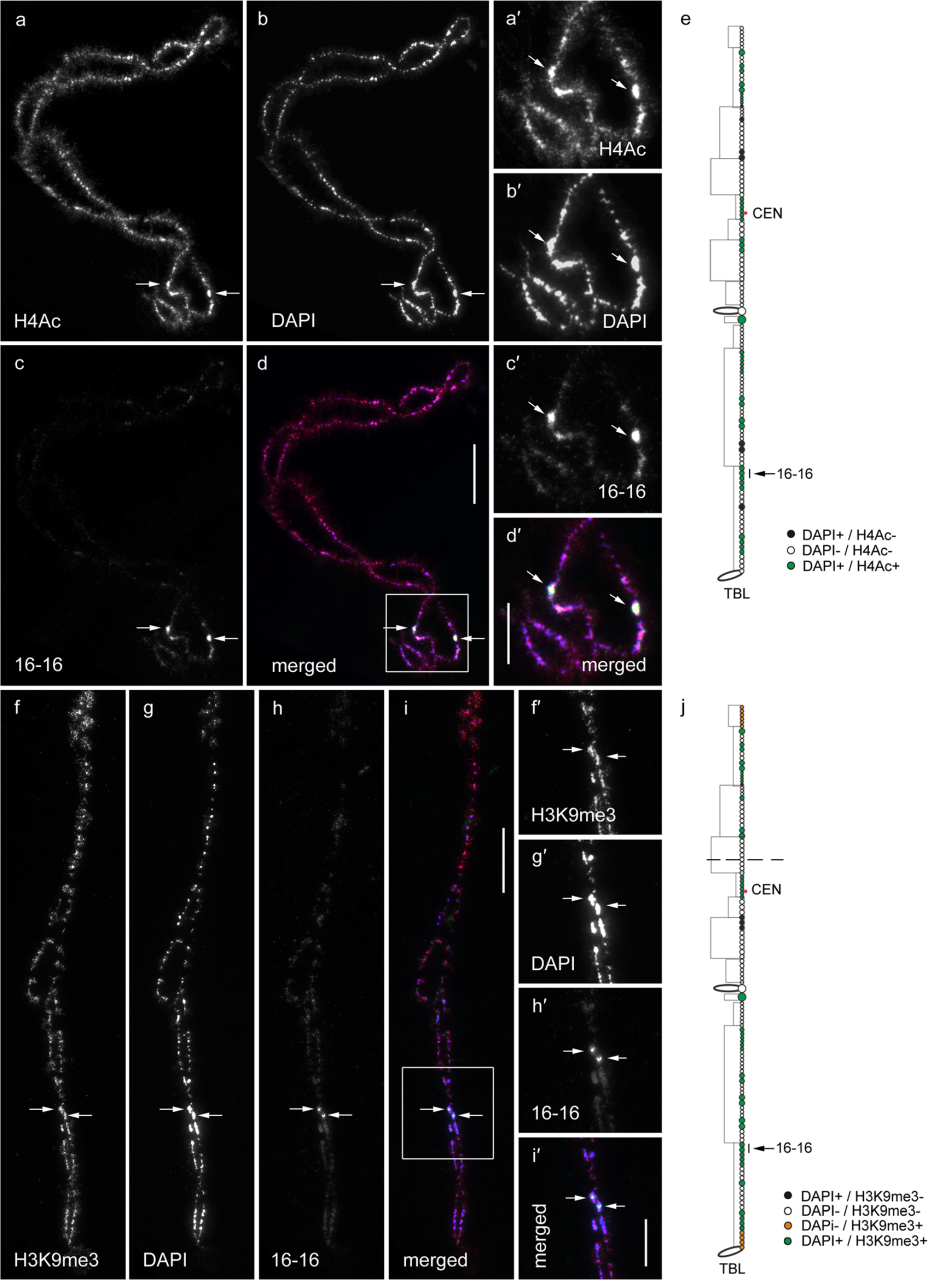
FISH with the DNA-probe to individual marker chromomere on GGA1 q-arm after immunodetection of H4Ac and H3K9me3. **a** – immunodetection of H4Ac on GGA1. **f** – immunodetection of H3K9me3 on the fragment of GGA1 q-arm. **b**, **g** – DAPI staining. **c**, **h** – FISH with the DNA probe #16–16 to individual marker chromomere of GGA1. **d**, **i** – merged **a**-**c** and **f**-**h** images, correspondingly (immunostaining – red, DAPI – blue, FISH signals – green). **a′**-**d′**, **f′**-**i′** – enlarged areas of panels **a**-**d** and **f**-**i** framed on **d** and **f**. Arrows indicate FISH signals. Scale bars: **a**-**d**, **f**-**i** – 20 μm; **a′**-**d′**, **f′**-**i′** – 10 μm. **e**, **j** – maps of H4Ac and H3K9me3 distribution on GGA1, correspondingly. DAPI-positive chromomeres – white circles, DAPI-negative chromomeres – black circles, DAPI-positive chromomeres enriched with H4Ac or H3K9me3 – green circles, DAPI-negative chromomeres enriched with H4Ac or H3K9me3 – orange circles. Arrows indicate position of the chromomere hybridized with the DNA probe #16–16, dashed line (**j**) indicates border of the GGA1 fragment (**f** - **i**). CEN – centromere position, TBL – telomere bow-like loops

To relate the epigenetic status of chromatin with a DNA sequence context of lampbrush chromomeres we combined immunofluorescence and FISH with seven DNA-probes obtained by mechanical microdissection of individual chromomeres (Fig. 7, Supplementary Fig. 2, 3). DNA sequences of microdissected samples were previously deciphered, mapped to the particular genomic regions and bioinformatically analysed [21, 22]. The first chromomere analyzed was large (about 5 Mb in size), DAPI-positive, marker chromomere on the q-arm of GGA1 for which a DNA probe #16–16 was obtained [21]. Immuno-FISH with the DNA probe #16–16 revealed that this chromomere combines conflicting epigenetic modifications: it was highly enriched with both H4Ac (Fig. 7 a-e) and H3K9me3 (Fig. 7 f-j) and according to the established map of 5mC distribution contained highly methylated DNA (Fig. 1 c-c′′). Following the genome context analysis, this chromomere is gene-poor and enriched with repetitive sequences including simple repeats and dispersed retrotransposons such as CR1 repeat of LINE family [21]. Juxtaposition of DNA sequences of the chromomere #16–16 to large scale chromatin compartments (A/B-compartments), identified by Hi-C technique in the interphase nucleus of chicken embryonic fibroblasts [27], demonstrated that they belong to B-compartment [22].

A combination of H3K9me3 and 5mC enrichment with H4Ac depletion was revealed in two individual chromomeres analyzed by immuno-FISH: large DAPI-positive chromomere (about 5 Mb) bearing a marker lumpy loop on GGA3q (LL3) (DNA probes #16–6 and #16–4) [21] and a small DAPI-negative chromomere (2.4 Mb in size) in GGA4q (DNA probe #17) [22] (Supplementary Fig. 2, 3 f-j). Two DNA probes #16–6 and #16–4 obtained by microdissection of LL3-bearing chromomere on GGA3q were hybridized after immunodetection of H4Ac and H3K9me3, correspondingly. Both DNA probes hybridized to LL3-bearing chromomere and to two-three neighboring chromomeres (Supplementary Fig. 2). LL3-bearing chromomere demonstrated depletion with H4Ac (Supplementary Fig. 2 a-e) and enrichment with anti-H3K9me3 (Supplementary Fig. 2 f-j). On the map of 5mC distribution LL3-bearing chromomere is also marked as highly methylated (Fig. 3 c-c′′). FISH with the DNA probe #17 to GGA4q after immunodetection of H3K9me3 revealed moderate (but not bright) labeling of the hybridized chromomere (Supplementary Fig. 3 f-i, f′′′-i′′′). The position of this chromomere on the maps corresponds to 5mC-rich chromomere which is not enriched with H4Ac (Fig. 4 a′′′, c′′). DNA sequences from the microdissected samples #16–6 and #17 were shown to be enriched with repeats and for 70–75% correspond to B-compartment in chicken interphase nucleus [21, 22]. Thus gene-poor and repeat-rich chromomeres studied here (GGA1 sample #16–16, GGA3 sample #16–6, GGA4 sample #17) have different epigenetic status: closed in chromomeres #16–6 and #17 and mixed in #16–16. Interestingly, samples #16–6 and #17 demonstrate lower density of interspersed repeats in comparison to the sample #16–16.

Individual chromomeres identified by three DNA probes (#3, #6 and #18) to GGA4 demonstrated different combination of epigenetic modifications. The DNA probe #3 hybridized with a group of three-four neighboring DAPI-negative chromomeres near the termini of GGA4q. Distal chromomere of the group demonstrated bright labeling with anti-H4Ac whereas proximal chromomeres were faintly labeled (Supplementary Fig. 3 a-e, a′′-d′′). All hybridyzed chromomeres map to highly methylated cluster which is not enriched with H3K9me3 (Fig. 4 b′′′, c′′). DNA sequences of the sample #3 occupy 2.7 Mb region on GGA4 sequence assembly and demonstrate uneven gene enrichment, which is higher to the distal part of the region. 70% of #3 sample DNA sequences were attributed to A-compartment in chicken interphase nucleus [22]. Thus bright anti-H4Ac labeling of the distal chromomere hybridizing with the DNA probe #3 correlates with gene enrichment in the genomic region occupied by DNA sequences of the sample #3 .

The DNA probe #6 to a small DAPI-positive chromomere (1.5 Mb in size) in the terminal part of GGA4q hybridized to a chromomere faintly labeled with H3K9me3 (Supplementary Fig. 3 f-j, f′-i′′). On the maps of epigenetic modifications distribution, this chromomere was marked as enriched with both H4Ac and 5mC (Fig. 4 a′′′, c′′). The DNA probe #18 to a DAPI-negative chromomere (2.4 Mb in size) from the proximal part of GGA4q [22] hybridized to a single loose chromomere, which also can be stretched to three tiny chromomeres (Supplementary Fig. 3 a-e, a′-d′). Chromomeres hybridized with the DNA probe #18 were not enriched with H4Ac, although the bases of the lateral loops extending from these chromomeres were H4Ac-rich (Supplementary Fig. 3 a, d, a′, d′). According to our maps, chromomeres in this region are not enriched with H3K9me3 but contain highly methylated DNA (Fig. 4 a′′′, c′′). Following the DNA sequence analysis samples #6 and #18 correspond to regular chromomeres with mixed genomic context (comprised both gene-reach/repeat-poor and gene-poor/repeat-rich DNA) [22].

## Discussion

Here we described in detail the distribution of H4Ac, H3K9me3 and 5mC along the axes of chicken lampbrush chromosomes GGA1–4, Z and W. The brightest chromomeres were mapped on cytological chromomere-loop maps reflecting DAPI-staining pattern. One of the most interesting findings is that in chicken lampbrush macrochromosomes the majority of chromomeres brightly stained with DAPI comprise modifications of both transcriptionally repressed (5mC and H3K9me3) and active (H4Ac) chromatin. We argue that anti-H4Ac reveals transcriptionally-active microloops on the surface of chromomeres. Such small loops forming a rosette structures were observed by electron microscopy of Miller spreads of chicken lampbrush chromosomes as well as after simultaneous immunodetection of H4Ac and elongating form of RNA-polymerase II [3]. We suggest that chromomeres bearing conflicting epigenetic landmarks comprise DNA sequences that generally should be repressed but remain transcriptionally active during the lampbrush stage. Examples of the chromomeres combining conflicting histone modifications are regions surrounding the centromeres in all studied chicken chromosomes, except for GGA3, and terminal region of GGAZq occupied by a cluster of Z-macrosatellite. Using immuno-FISH with the chromomere-specific DNA probes obtained by microdissection [21] we revealed that marker chromomere #16–16 on GGA1q also combines such conflicting epigenetic modifications. Enrichment of the chromomere #16–16 with H4Ac correlates with the high density of retrotransposons suggesting their potential transcription during the lampbrush stage.

Some chromomeres and chromosomal regions are enriched only with the markers of repressed chromatin (5mC and H3K9me3) and depleted for H4Ac. Such epigenetic status is typical for chromomeres containing certain tandem repeats, for instance terminal chromomeres of GGA1p/q and GGA2q containing PO41 repeat; chromomeres containing non-transcribing clusters of CNM repeat on GGA3q and GGAW (chromomere 4); as well as chromomeres containing *Eco*RI- and *Xho*I- repeats in GGAW (chromomeres 1, 3 and 5). Similar combination of epigenetic modifications was found in repeat-rich chromomeres #16–6 on GGA3q and #17 on GGA4q.

Another interesting finding is that cytosine methylation pattern on chicken lampbrush chromosomes significantly differs from that on mitotic metaphase chromosomes. In chicken mitotic metaphase karyotype, hypermethylated regions were generally restricted to constitutive heterochromatin [28], whereas in lampbrush chromosomes numerous chromosomal regions were enriched with 5mC. For instance, in metaphase GGA1 only the pericentromere region is highly methylated [28], while in lampbrush GGA1 clusters of highly methylated chromomeres are also found in many other regions. In completely heterochromatic metaphase chromosome W hypermethylated DNA is restricted to the subtelomere region [28], whereas at the lampbrush stage DNA of all lampbrush chromomeres of chromosome W is highly methylated. These observations suggest that meiotic diplotene chromosomes demonstrate a specific DNA methylation pattern different from that in mitotic metaphase chromosomes. On the other hand, the difference in methylation pattern may be due to dissimilar DNA denaturing conditions used for meiotic lampbrush and mitotic metaphase chromosomes.

## Conclusions

Here we described in detail the epigenetic landscape of chicken meiotic chromosomes 1–4, Z and W at the lampbrush stage. On the base of established cytological chromomere-loop maps we developed maps reflecting the distribution of epigenetic modifications (5mC, H3K9me3 and H4Ac) on GGA1–4, GGAZ and GGAW. The developed maps can be used to establish a correlation between epigenetically different chromatin domains with their transcriptional activity, level of compaction and 3D-organization in interphase nucleus. We demonstrated that a combination of immunofluorescence staining and fluorescence in situ hybridization allows to relate the epigenetic status and the DNA sequence context of individual chromomeres. As a probe one can use DNA from dissected material, probes to repetitive elements, cloned DNA-fragments or region-specific oligonucleotide paints.

## Methods

### Lampbrush chromosome preparations

Chicken lampbrush chromosomes were prepared according to previously described procedure [29, 30] with minor modifications. The oocytes with a diameter from 1 to 2.5 mm were dissected from the ovary and placed in a cooled “5:1” medium (83 mM KCl, 17 mM NaCl, 6.5 mM Na_2_HPO_4_, 3.5 mM KH_2_PO_4_, 1 mM MgCl_2_, 1 mM DTT, pH 7.2). Oocytes and nuclei were manipulated under a Leica MZ16 stereomicroscope. To release the nucleus, oocyte membrane was broken with the help of tungsten needles. The isolated nucleus was washed with a hypotonic “1/4” medium (“5:1” medium diluted 4 times and containing 0.1% formaldehyde, 1 mM MgCl_2_) and transferred to the slide mounted chamber filled with “1/4” medium. The nuclear envelope was removed by thin tungsten needles. Preparations were centrifuged for 20 min at 4000 rpm and + 4 °C, fixed in 2% formaldehyde in PBS for 20 min, dehydrated in ethanol series (50, 70%) and left in 70% ethanol overnight at + 4 °C. The animal studies received an approval of the Ethical committee of Saint-Petersburg State University (#131–03-2, 14.03.2016).

### Immunofluorescent staining

Immunostaining of chicken lampbrush chromosomes was carried out as previously described [12] with the following primary antibodies: rabbit polyclonal antibodies against H4Ac (06–866, Millipore), rabbit polyclonal antibodies against H3K9me3 (8898, Abcam), and mouse antibodies against 5mC (ab51552, Abcam). The slides were rehydrated in a series of ethanol (50, 30%) and then in PBS for 5 min. To reveal the distribution of 5mC, chromosomes were denatured in 2 M HCl for 60–90 min followed by thrice 5 min wash in PBS. Then preparations were blocked with 0.5% blocking reagent (Calbiochem) in PBS or with 1% BSA in PBS in case of 5mC. Primary and secondary antibodies were diluted in the blocking solution according to manufacturer’s recommendations. Following secondary antibodies were used: Alexa-488 conjugated goat anti-rabbit IgG (Molecular Probes) and Cy3 conjugated goat anti-mouse IgG (Jackson Immuno Research Laboratories). All incubations were performed in a humidity chamber at RT for 60 min. After incubation with antibodies, preparations were washed thrice in PBS with 0.02% Tween 20 for 5 min at RT. Finally, the slides were dehydrated in ethanol series (50, 70, 96%) for 5 min, air dried and mounted in antifade solution containing 1 μg/ml DAPI (4,6-diamidino-2-phenylindole).

### Probe labeling

FISH probes used in this study were generated from chromomeres mechanically microdissected from preparations of chicken lampbrush chromosomes [21, 22]. Dissected chromosomal material was primary amplified by DOP-PCR [31]. DNA probes were generated via DOP-PCR reamplification and PCR-labeling with biotin or digoxigenin. Here we used DNA probes developed from dissected chromomeres of chicken lampbrush macrochromosomes GGA1 (DNA probe #16–16), GGA3 (DNA probes #16–4, #16–6) [21] and GGA4 (DNA probes ## macro3, 6, 17, 18) [22]. Biotinylated oligonucleotide probe CNMpos [17] was used to localize the CNM repeat.

### Fluorescence in situ hybridization

Fluorescence in situ hybridization (FISH) was performed on selected lampbrush chromosome preparations after immunostaining according to a DNA/(DNA + RNA) hybridization protocol [7]. Hybridization mixture contained 20 ng/μl DNA probe, 50% formamide, 10% dextran sulfate, 2 × SSC (0.3 M NaCl, 30 mM Na_3_C_6_H_5_O_7_), 1 μg/μl salmon sperm DNA and deionized water for PCR generated DNA probes; for oligonucleotide probe formamide concentration was decreased to 42%. Chromosomes and DNA-probes were denatured simultaneously for 5 min at 78 °C followed by overnight hybridization in a humidity chamber at 37 °C for PCR generated DNA probes or at room temperature for oligonucleotide probe. The slides were washed in two changes of 0.2 × SSC at 60 °C after hybridization with PCR generated DNA probes and two changes of 2 × SSC at 45 °C or in three changes of 2xSCC at 37 °C after FISH with oligonucleotide probe. DNA probes labeled with biotin and digoxigenin were detected with Cy3- or Alexa488- conjugated avidin (Jackson Immuno Research Laboratories) and Cy3-conjugated anti-digoxigenin antibody (Jackson Immuno Research Laboratories) correspondingly. Biotinylated anti-avidin (Vectorlabs) and Cy3-conjugated goat anti-mouse antibody (Jackson Immuno Research Laboratories) were used to amplify hybridization signals of the corresponding DNA probes. After FISH slides were mounted in antifade solution containing DAPI (1 μg/ml).

### Fluorescent microscopy

The slides were analyzed using Leica DM4000 and/or DM6000 fluorescence microscopes (Leica-Microsystems) equipped with a monochrome high-sensitivity digital CCD camera with a resolution of 1.3 megapixels and the appropriate set of filter cubes. The morphology of lampbrush chromosomes was analyzed in a phase contrast mode.

### Image analysis and mapping of epigenetic modifications

The intensity of immunofluorescence signals on obtained microphotographs was evaluated visually as for the maps reflecting DAPI staining pattern on chicken lampbrush chromosomes [6]. The most brightly stained chromomeres consistently demonstrating bright fluorescence were mapped to the established cytological chromomere-loop maps of lampbrush macrochromosomes GGA1, GGA2, GGA3, GGA4 and GGAZW reflecting a pattern of DAPI staining [6, 15]. For each of the examined epigenetic modifications, from three to six individual corresponding lampbrush chromosomes were analyzed. FISH-positive chromomeres were also mapped to the corresponding cytological chromomere-loop maps; their genomic positions were previously verified based on high-throughput sequencing data [21, 22].

## Supplementary information

**Additional file 1: Supplementary Figure1.** Positioning of chromomere 4 on GGAW after immunodetection of H4Ac. FISH with oligonucleotide probe to CNM repeat after immunostaining of GGAW with antibodies against H4Ac. a – H4Ac, a’ – DAPI, a” – FISH with oligonucleotide probe to CNM repeat (green) merged with H4Ac (red) and DAPI (blue). Numbers (1–7) indicate individual chromomeres of GGAW from free terminus to chiasma with the chromosome Z. Scale bar – 10 μm. **Supplementary Figure 2.** FISH with the DNA-probes to the chromomere bearing LL32 on GGA3 q-arm after immunodetection of H4Ac and H3K9me3. a – immunodetection of H4Ac on GGA3, f –immunodetection of H3K9me3 on the fragment of GGA3. b, g – DAPI staining. c, h – FISH with the DNA probes #16–6 and #16–4 to the chromomere bearing LL32 correspondingly. d, i – merged a-c and f-h images correspondingly (immunostaining – red, DAPI – blue, FISH signals – green). a’-d’, f’-i’ – enlarged areas of panels a-d and f-i, framed on d and i. Arrows indicate FISH signals. Scale bars on panels a-d, f-i – 20 μm, on panels a’-d’, f’-i’ – 10 μm. e, j – maps of H4Ac and H3K9me3 distribution on GGA3 correspondingly. DAPI-positive chromomeres – white circles, DAPI-negative chromomeres – black circles, DAPI-positive chromomeres enriched with H4Ac or H3K9me3 – green circles, DAPI-negative chromomeres enriched with H4Ac or H3K9me3 – orange circles. Arrows indicate chromomeres hybridized with the corresponding DNA probes. Dashed line (j) indicates border of the GGA3 fragment (f-i). CEN – centromere position, TBL – telomere bowlike loops. LL32 – lumpy loop 32. **Supplementary Figure 3.** FISH with the DNA-probes to individual chromomeres of GGA4 after immunodetection of H4Ac or H3K9me3. a – immunodetection of H4Ac on GGA4. f – immunodetection of H3K9me3 on GGA4. b, g – DAPI staining. c, h – FISH with the DNA probes #3 + #18 and #6 + #17 to individual chromomeres of GGA4 q-arm correspondingly. d, i – merged a-c and f-h images correspondingly (immunostaining – red, DAPI – blue, FISH signals – green). Enlarged areas of panels a-d and f-i, framed on d and i correspondingly: a’-d’ – DNA probe #18; a”-d” – DNA probe #3, f’ - i’, f”-i” – DNA probe #6; f”‘-i”‘– DNA probe #17. Arrows indicate FISH signals, arrowhead (h) – position of chromosome termini. Scale bars on panels a-d, f-i – 20 μm, on panels a’-d”, f’-i”‘– 10 μm. e, j – maps of H4Ac and H3K9me3 distribution on GGA4 correspondingly. DAPI-positive chromomeres – white circles, DAPI-negative chromomeres – black circles, DAPI-positive chromomeres enriched with H4Ac or H3K9me3 – green circles, DAPI-negative chromomeres enriched with H4Ac or H3K9me3 – orange circles. Arrows indicate chromomeres hybridized with the corresponding DNA probes. CEN – centromere position, GITERA – giant terminal RNP aggregates.

## Data Availability

All data generated or analysed during this study are included in this published article and its supplementary information files.

## Abbreviations

5mC: 5-methylcytosine
BAC: Bacterial artificial chromosome
CNM: Chicken nuclear-membrane-associated repeat
CR1: Chicken repeat 1
DAPI: 4′,6-diamidino-2-phenylindole
DOP-PCR: Degenerate oligonucleotide-primed PCR
FISH: Fluorescence in situ hybridization
GGA: Chicken (*Gallus gallus*) chromosome
GITERA: Giant terminal RNP aggregates
H3K27me3: Histone H3 trimethylated at lysine 27
H3K9me3: Histone H3 trimethylated at lysine 9
H4Ac: Hyperacetylated histone H4
LINE: Long interspersed elements
LL2: Lumpy loop 2
LL32: Lumpy loop 32
p-arm: Short arm of a chromosome
PBS: Phosphate-buffered saline
PO41: Pattern of 41 repeat
q-arm: Long arm of a chromosome
SM: Spaghetti marker
SSC: Saline sodium citrate buffer
TBL: Telomere bow-like loops

## References

[1] Callan HG (1986). Lampbrush chromosomes.

[2] Morgan GT (2002). Lampbrush chromosomes and associated bodies: new insights into principles of nuclear structure and function. Chromosom Res.

[3] Gaginskaya E, Kulikova T, Krasikova A (2009). Avian Lampbrush chromosomes: a powerful tool for exploration of genome expression. Cytogenet Genome Res..

[4] Callan HG, Lloyd L (1960). Lampbrush chromosomes of crested newts Triturus cristatus (Laurenti). Philos Trans R Soc Lond Ser B Biol Sci.

[5] Macgregor HC (2012). Chromomeres revisited. Chromosom Res.

[6] Galkina S, Deryusheva S, Fillon V, Vignal A, Crooijmans R, Groenen M (2006). FISH on avian lampbrush chromosomes produces higher resolution gene mapping. Genetica..

[7] Zlotina A, Krasikova A, Liehr T (2017). FISH in Lampbrush Chromosomes. Fluoresc Situ Hybrid FISH.

[8] Hock R, Moorman A, Fischer D, Scheer U (1993). Absence of somatic histone H1 in oocytes and Preblastula embryos of Xenopus laevis. Dev Biol.

[9] Sommerville J, Baird J, Turner BM (1993). Histone H4 acetylation and transcription in amphibian chromatin. J Cell Biol.

[10] Angelier N, Bonnanfant-Ja’is ML, Moreau N, Gounon P, Lavaud A (1986). DNA methylation and RNA transcriptional activity in amphibian lampbrush chromosomes. Chromosoma..

[11] Krasikova AV, Kulikova TV (2017). Distribution of heterochromatin markers in lampbrush chromosomes in birds. Russ J Genet.

[12] Krasikova A, Daks A, Zlotina A, Gaginskaya E (2009). Polymorphic heterochromatic segments in Japanese quail microchromosomes. Cytogenet Genome Res..

[13] Warren WC, Hillier LW, Tomlinson C, Minx P, Kremitzki M, Graves T (2017). A New Chicken Genome Assembly Provides Insight into Avian Genome Structure. G3 Genes Genomes Genetics.

[14] Krasikova A, Deryusheva S, Galkina S, Kurganova A, Evteev A, Gaginskaya E (2006). On the positions of centromeres in chicken lampbrush chromosomes. Chromosom Res.

[15] Zlotina A, Galkina S, Krasikova A, Crooijmans RPMA, Groenen MAM, Gaginskaya E (2012). Centromere positions in chicken and Japanese quail chromosomes: de novo centromere formation versus pericentric inversions. Chromosom Res.

[16] Galkina S, Fillon V, Saifitdinova A, Daks A, Kulak M, Dyomin A (2017). Chicken microchromosomes in the Lampbrush phase: a cytogenetic description. Cytogenet Genome Res..

[17] Deryusheva S, Krasikova A, Kulikova T, Gaginskaya E (2007). Tandem 41-bp repeats in chicken and Japanese quail genomes: FISH mapping and transcription analysis on lampbrush chromosomes. Chromosoma..

[18] Zlotina A, Galkina S, Krasikova A, Crooijmans RPMA, Groenen MAM, Gaginskaya E (2010). Precise centromere positioning on chicken chromosome 3. Cytogenet Genome Res.

[19] Krasikova AV, Vasilevskaya EV, Gaginskaya ER (2010). Chicken lampbrush chromosomes: transcription of tandemly repetitive DNA sequences. Russ J Genet.

[20] Bellott DW, Skaletsky H, Cho T-J, Brown L, Locke D, Chen N (2017). Avian W and mammalian Y chromosomes convergently retained dosage-sensitive regulators. Nat Genet.

[21] Zlotina A, Kulikova T, Kosyakova N, Liehr T, Krasikova A (2016). Microdissection of lampbrush chromosomes as an approach for generation of locus-specific FISH-probes and samples for high-throughput sequencing. BMC Genomics.

[22] Zlotina A, Maslova A, Pavlova O, Kosyakova N, Al-Rikabi A, Liehr T (2020). New insights into Chromomere organization provided by Lampbrush chromosome microdissection and high-throughput sequencing. Front Genet.

[23] Hori T, Suzuki Y, Solovei I, Saitoh Y, Hutchison N, Ikeda J-E (1996). Characterization of DNA sequences constituting the terminal heterochromatin of the chicken Z chromosome. Chromosom Res.

[24] Solovei I, Ogawa A, Naito M, Mizuno S, Macgregor H (1998). Specific chromomeres on the chicken W lampbrush chromosome contain specific repetitive DNA sequence families. Chromosom Res.

[25] Itoh Y, Mizuno S (2002). Molecular and cytological characterization of SspI-family repetitive sequence on the chicken W chromosome. Chromosom Res.

[26] Komissarov AS, Galkina SA, Koshel EI, Kulak MM, Dyomin AG, O’Brien SJ (2018). New high copy tandem repeat in the content of the chicken W chromosome. Chromosoma..

[27] Fishman V, Battulin N, Nuriddinov M, Maslova A, Zlotina A, Strunov A (2019). 3D organization of chicken genome demonstrates evolutionary conservation of topologically associated domains and highlights unique architecture of erythrocytes’ chromatin. Nucleic Acids Res.

[28] Schmid M, Smith J, Burt DW, Aken BL, Antin PB, Archibald AL (2015). Third report on chicken genes and chromosomes 2015. Cytogenet Genome Res..

[29] Kropotova EV, Gaginskaya ER (1984). Lampbrush chromosomes from the Japanese quail oocytes. Tsitologiia..

[30] Solovei I, Gaginskaya E, Allen T, Macgregor HC (1992). A novel structure associated with a lampbrush chromosome in the chicken, Gallus domesticus. J Cell Sci.

[31] Telenius H, Carter NP, Bebb CE, Nordenskjo M, Ponder BAJ, Tunnacliffe A (1992). Degenerate oligonucleotide-primed PCR: General amplification of target DNA by a single degenerate primer. Genomics.

