# Supplementary material for: Mapping epigenetic modifications on chicken lampbrush chromosomes
Source: Mol Cytogenet. 2020 Aug 3;13:32. doi: 10.1186/s13039-020-00496-0 (PMC7397634; doi:10.1186/s13039-020-00496-0)
Supplement: Supplementary file 1 — Additional file 1: Supplementary Figure1. Positioning of chromomere 4 on GGAW after immunodetection of H4Ac. FISH with oligonucleotide probe to CNM repeat after immunostaining of GGAW with antibodies against H4Ac. a – H4Ac, a’ – DAPI, a” – FISH with oligonucleotide probe to CNM repeat (green) merged with H4Ac (red) and DAPI (blue). Numbers (1–7) indicate individual chromomeres of GGAW from free terminus to chiasma with the chromosome Z. Scale bar – 10 μm. Supplementary Figure 2. FISH with the DNA-probes to the chromomere bearing LL32 on GGA3 q-arm after immunodetection of H4Ac and H3K9me3. a – immunodetection of H4Ac on GGA3, f –immunodetection of H3K9me3 on the fragment of GGA3. b, g – DAPI staining. c, h – FISH with the DNA probes #16–6 and #16–4 to the chromomere bearing LL32 correspondingly. d, i – merged a-c and f-h images correspondingly (immunostaining – red, DAPI – blue, FISH signals – green). a’-d’, f’-i’ – enlarged areas of panels a-d and f-i, framed on d and i. Arrows indicate FISH signals. Scale bars on panels a-d, f-i – 20 μm, on panels a’-d’, f’-i’ – 10 μm. e, j – maps of H4Ac and H3K9me3 distribution on GGA3 correspondingly. DAPI-positive chromomeres – white circles, DAPI-negative chromomeres – black circles, DAPI-positive chromomeres enriched with H4Ac or H3K9me3 – green circles, DAPI-negative chromomeres enriched with H4Ac or H3K9me3 – orange circles. Arrows indicate chromomeres hybridized with the corresponding DNA probes. Dashed line (j) indicates border of the GGA3 fragment (f-i). CEN – centromere position, TBL – telomere bowlike loops. LL32 – lumpy loop 32. Supplementary Figure 3. FISH with the DNA-probes to individual chromomeres of GGA4 after immunodetection of H4Ac or H3K9me3. a – immunodetection of H4Ac on GGA4. f – immunodetection of H3K9me3 on GGA4. b, g – DAPI staining. c, h – FISH with the DNA probes #3 + #18 and #6 + #17 to individual chromomeres of GGA4 q-arm correspondingly. d, i – merged a-c and f-h images correspondingly (immunostai [file 13039_2020_496_MOESM1_ESM.pdf]

Electronic Supplementary Material

Mapping epigenetic modifications on chicken lampbrush chromosomes

Tatiana Kulikova[1], Anna Surkova[1], Anna Zlotina[1], Alla Krasikova[1]*

[1]Saint Petersburg State University, Saint-Petersburg, Russia

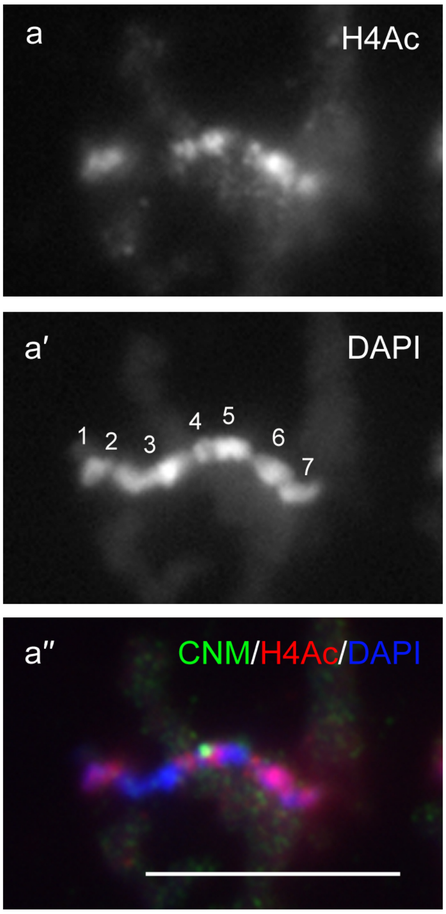
a
H4Ac
a′
DAPI
1
2
3
4
5
6
7
a″
CNM/H4Ac/DAPI

Supplementary Figure 1. Positioning of chromomere 4 on GGAW after immunodetection of H4Ac.

FISH with oligonucleotide probe to CNM repeat after immunostaining of GGAW with antibodies against H4Ac. **a** – H4Ac, **a′** – DAPI, **a″** – FISH with oligonucleotide probe to CNM repeat (green) merged with H4Ac (red) and DAPI (blue). Numbers (**1**-**7**) indicate individual chromomeres of GGAW from free terminus to chiasma with the chromosome Z.

Scale bar – 10 μm.

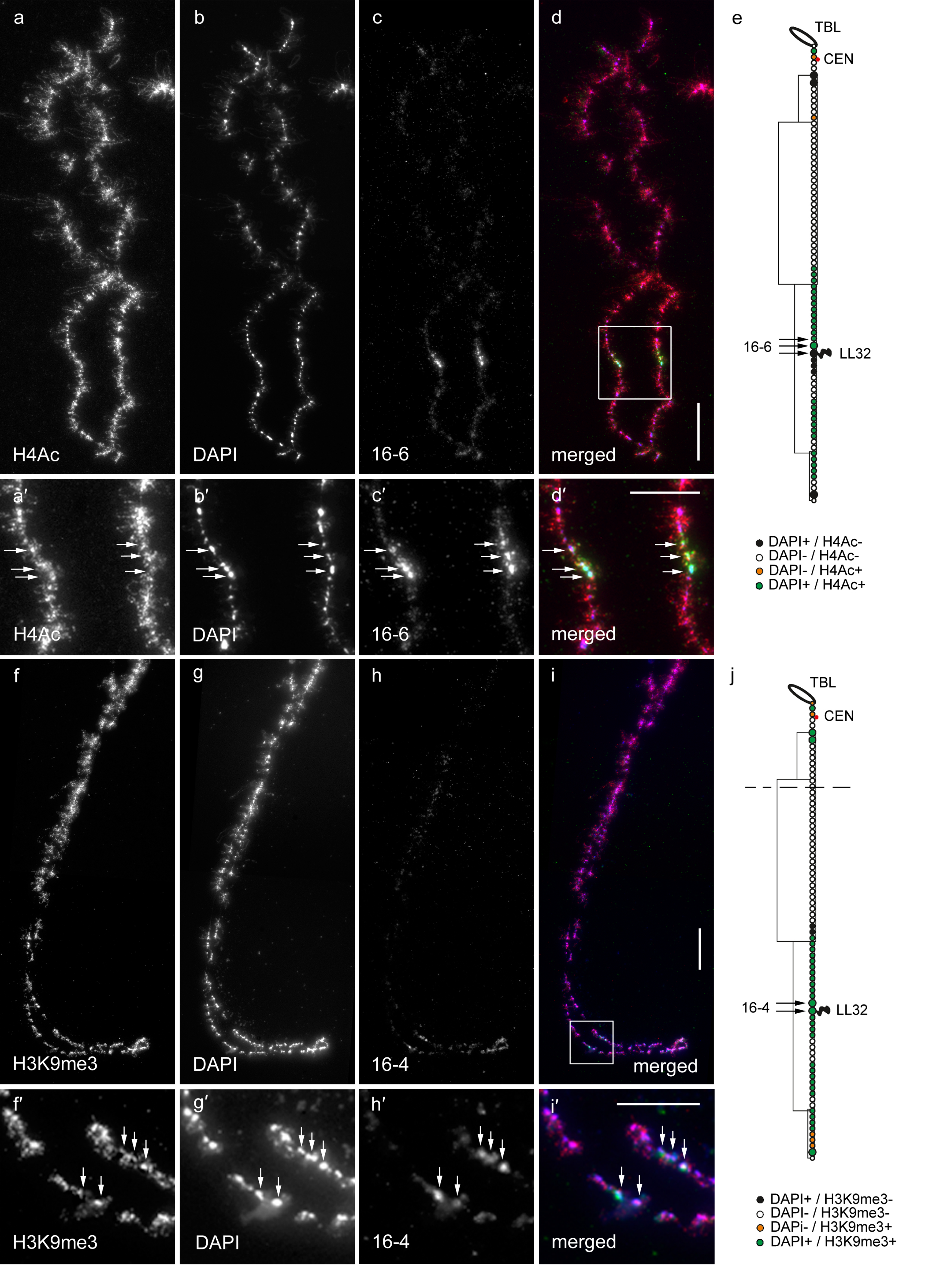

a
b
c
d
e
H4Ac
DAPI
16-6
merged
TBL
CEN
16-6
LL32
a′
b′
c′
d′
H4Ac
DAPI
16-6
merged
DAPI+ / H4Ac-
DAPI- / H4Ac-
DAPI- / H4Ac+
DAPI+ / H4Ac+
f
g
h
i
j
H3K9me3
DAPI
16-4
merged
TBL
CEN
16-4
LL32
f′
g′
h′
i′
H3K9me3
DAPI
16-4
merged
DAPI+ / H3K9me3-
DAPI- / H3K9me3-
DAPi- / H3K9me3+
DAPI+ / H3K9me3+

Supplementary Figure 2. **FISH with the DNA-probes to the chromomere bearing LL32 on GGA3 q-arm after immunodetection of H4Ac and H3K9me3.**

a – immunodetection of H4Ac on GGA3, **f** –immunodetection of H3K9me3 on the fragment of GGA3. **b**, **g** – DAPI staining. **c**, **h** – FISH with the DNA probes #**16-6** and #**16-4** to the chromomere bearing LL32 correspondingly. **d**, **i** – merged **a**-**c** and **f**-**h** images correspondingly (immunostaining – red, DAPI – blue, FISH signals – green). **a′**-**d′**, **f′**-**i′** – enlarged areas of panels **a**-**d** and **f**-**i**, framed on **d** and **i**. Arrows indicate FISH signals. Scale bars on panels **a**-**d**, **f**-**i** – 20 μm, on panels **a′**-**d′**, **f′**-**i′** – 10 μm. **e**, **j** – maps of H4Ac and H3K9me3 distribution on GGA3 correspondingly. DAPI-positive chromomeres – white circles, DAPI-negative chromomeres – black circles, DAPI-positive chromomeres enriched with H4Ac or H3K9me3 – green circles, DAPI-negative chromomeres enriched with H4Ac or H3K9me3 – orange circles. Arrows indicate chromomeres hybridized with the corresponding DNA probes. Dashed line (**j**) indicates border of the GGA3 fragment (**f**-**i**). CEN – centromere position, TBL – telomere bow-like loops. LL32 – lumpy loop 32.

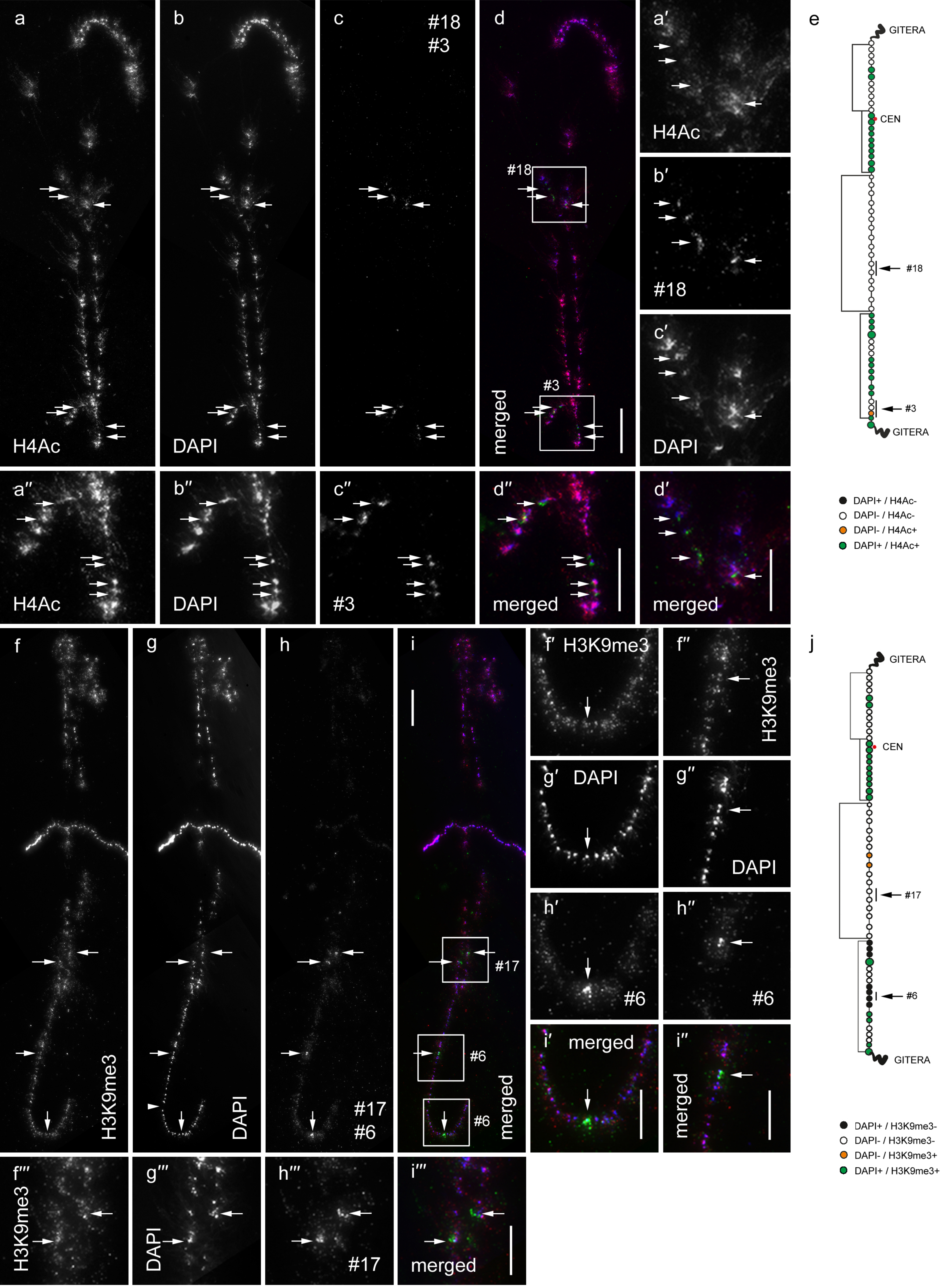

a
b
c
d
a′
e
#18
#3
H4Ac
DAPI
merged
#18
#3
GITERA
CEN
GITERA
#18
#3
DAPI
a″
b″
c″
d″
d′
H4Ac
DAPI
#3
merged
merged
DAPI+ / H4Ac-
DAPI- / H4Ac-
DAPI- / H4Ac+
DAPI+ / H4Ac+
f
g
h
i
f′ H3K9me3
f″
j
g′ DAPI
g″
h′
h″
i′ merged
i″
H3K9me3
DAPI
#17
#6
#17
#6
#6
merged
#6
#6
GITERA
CEN
#17
#6
GITERA
DAPI+ / H3K9me3-
DAPI- / H3K9me3-
DAPI- / H3K9me3+
DAPI+ / H3K9me3+
f‴
g‴
h‴
i‴
H3K9me3
DAPI
#17
merged

Supplementary Figure 3. FISH with the DNA-probes to individual chromomeres of GGA4 after immunodetection of H4Ac or H3K9me3. **a** – immunodetection of H4Ac on GGA4. **f** – immunodetection of H3K9me3 on GGA4. **b**, **g** – DAPI staining. **c**, **h** – FISH with the DNA probes **#3+#18** and **#6+#17** to individual chromomeres of GGA4 q-arm correspondingly. **d**, **i** – merged **a-c** and **f-h** images correspondingly (immunostaining – red, DAPI – blue, FISH signals – green). Enlarged areas of panels **a-d** and **f-i**, framed on **d** and **i** correspondingly: **a′-d′** – DNA probe **#18**; **a″-d″** – DNA probe **#3**, **f′ - i′**, **f″-i″** – DNA probe **#6**; **f‴-i‴** – DNA probe **#17**. Arrows indicate FISH signals, arrowhead (**h**) – position of chromosome termini. Scale bars on panels **a-d**, **f-i** – 20 μm, on panels **a′-d″**, **f′-i‴** – 10 μm. **e**, **j** – maps of H4Ac and H3K9me3 distribution on GGA4 correspondingly. DAPI-positive chromomeres – white circles, DAPI-negative chromomeres – black circles, DAPI-positive chromomeres enriched with H4Ac or H3K9me3 – green circles, DAPI-negative chromomeres enriched with H4Ac or H3K9me3 – orange circles. Arrows indicate chromomeres hybridized with the corresponding DNA probes. CEN – centromere position, GITERA – giant terminal RNP aggregates.
